# Review and Analysis of Modern Laser Beam Welding Processes

**DOI:** 10.3390/ma17184657

**Published:** 2024-09-23

**Authors:** Andrzej Klimpel

**Affiliations:** Welding Department, Faculty of Mechanical Engineering, Silesian University of Technology, Konarskiego 18A Str., 44-100 Gliwce, Poland; andrzej.klimpel@polsl.pl

**Keywords:** welding, laser beam, melt-in mode, keyhole mode, EXC3-4, LBW, LHW, GMA, MAG, HAZ, AI, quality, online quality monitoring

## Abstract

Laser beam welding is the most modern and promising process for the automatic or robotized welding of structures of the highest Execution Class, EXC3-4, which are made of a variety of weldable structural materials, mainly steel, titanium, and nickel alloys, but also a limited range of aluminum, magnesium, and copper alloys, reactive materials, and even thermoplastics. This paper presents a systematic review and analysis of the author’s research results, research articles, industrial catalogs, technical notes, etc., regarding laser beam welding (LBW) and laser hybrid welding (LHW) processes. Examples of industrial applications of the melt-in-mode and keyhole-mode laser welding techniques for low-alloy and high-alloy steel joints are analyzed. The influence of basic LBW and LHW parameters on the quality of welded joints proves that the laser beam power, welding speed, and Gas Metal Arc (GMA) welding current firmly decide the quality of welded joints. A brief review of the artificial intelligence (AI)-supported online quality-monitoring systems for LBW and LHW processes indicates the decisive influence on the quality control of welded joints.

## 1. Introduction

Laser beam welding (LBW) processes, which are powered by a single laser radiation generator feature, have not yet been fully used, given their great industrial potential, compared to conventional arc welding processes. In particular, their advantages include their high quality and welding efficiency; their ease of automation and especially robotization; production flexibility, namely the ability to simultaneously weld and perform surfacing, melting, heat treatment, or cutting; the high melting depths of laser-welded butt joints and a very narrow Heat-Affected Zone (HAZ); low deformation; and almost zero residual stress [1,2,3,4,5,6,7,8,9].

**Figure 1:**
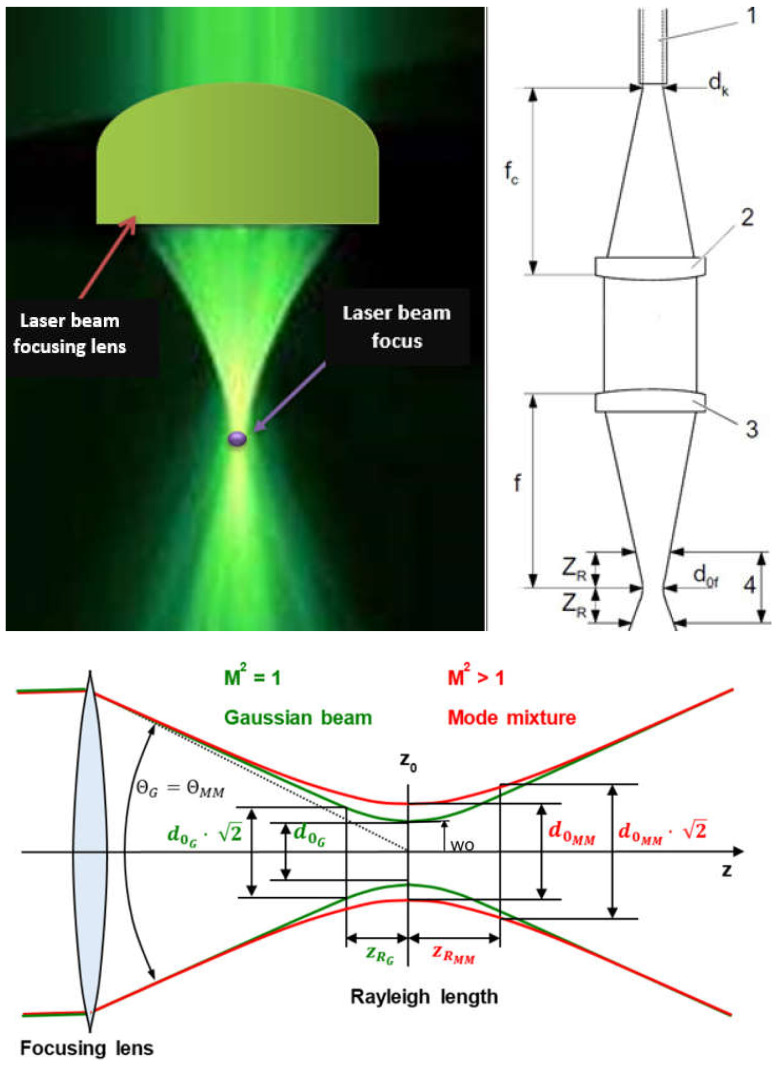
A view of the focused laser beam, TEM_00_, by the focusing lens over the surface of the welded joint and the schemes of a focused laser beam’s parameters: d_00_—the ideal focused spot diameter, Θ_G_—the beam deflection angle in the far field, w_0_—the radius of the laser beam constriction (diameter of the focus d_OG_ or d_0f_), 1—the laser beam, 2—the collimator, 3—the focusing lens, 4—the double Rayleigh length (depth-of-focus), d_k_—the diameter of the optical fiber core, f_c_—the focal length of the collimator, f—the focal length of the focusing lens, ZR—the Rayleigh length [1,8].

**Figure 2:**
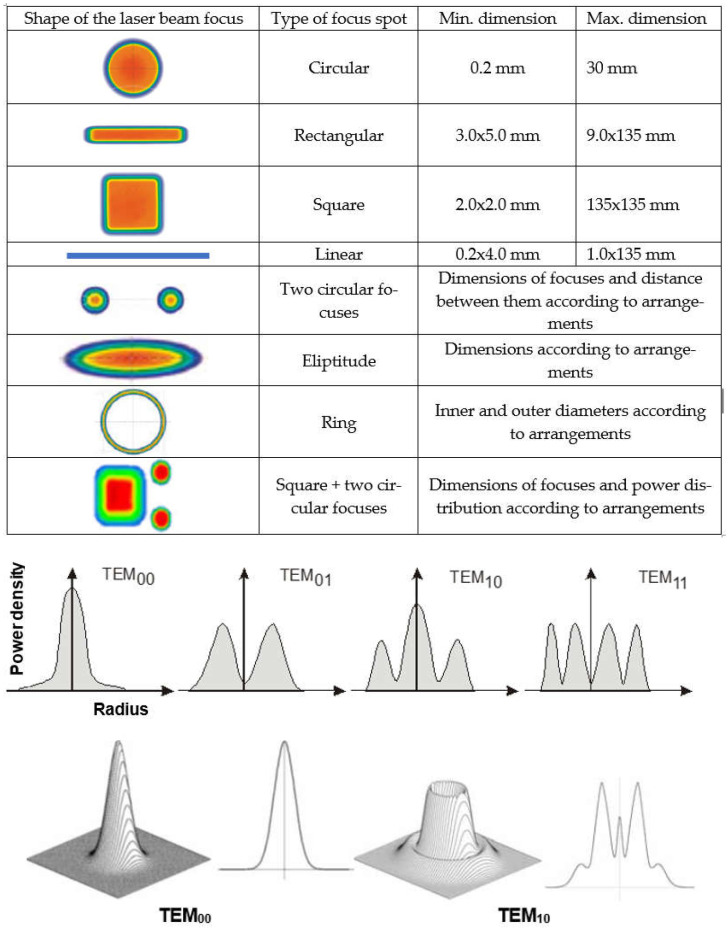
The shape of the laser beam focuses of modern solid-state lasers and their optical systems, and typical energy distributions at the focus of a laser beam—TEM (Transverse Electromagnetic Modes) [1,8].

**Figure 3:**
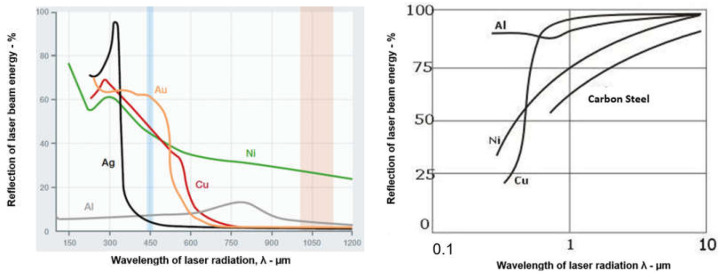
Effect of the wavelength of laser radiation λ—µm, on the reflection of laser beam energy from the surface of metal objects made of steel, Ni, Al, Ag, Au, and Cu [1].

The laser beam, which passes from the laser generator to the welded joint through a system of apertures, mirrors, and optical elements or optical fibers and optical systems, is focused on the weld area of the joint. The laser beam, however, cannot be focused at a point; hence, the focusing area is in the form of a tapered cylinder, as shown in Figure 1. The shape of the laser beam focus, which is formed by modern solid-state lasers and optical systems, makes it possible to obtain a laser beam focus with a cross-sectional shape: rectangular, square, circular, and elliptical, or even close to linear and multi-focus shapes. This allows versatility in all possible welding engineering processes, as shown in Figure 2. The laser beam incident on the surface of the welded joint area is intensely reflected and, depending on the type of parent material of the joint and the condition of its surface, the absorption coefficient of the laser radiation energy is only 1.0–5.0% for CO_2_ lasers, and for solid-state Nd/YAG, fiber, disk, and diode lasers, it is of the order of 2.0–50.0%, as shown in Figure 3 [1,5,6,7,8,9]. Thus, the technological and economic efficiency of a laser welding process depends on the quality and power of the laser beam and on the absorption coefficient of the laser beam’s energy on the surface of the welded joint. Laser beam light is reflected most intensively by aluminum, magnesium, and copper and their alloys, which is what makes its technological weldability difficult, as shown in Figure 3.

The absorption coefficient of the laser beam also depends on the temperature of the welded workpiece and the surface roughness; it increases to nearly 90% once the melting temperature of the welded material is exceeded, and almost all the laser radiation energy is absorbed once the evaporation temperature is exceeded. The absorption of laser radiation also depends on the power density of the laser beam. For low-power CO_2_ laser radiation, with a wavelength of 10.6 µm, only ~1.0% of the laser beam energy is absorbed by the polished aluminum and copper surfaces of welded joints, while as much as 40% is absorbed by the rough surfaces of corrosion-resistant austenitic steels. Once the threshold for the laser beam power density is exceeded—which for carbon steels, low-alloy steels, and high-alloy steels is about 1.5 × 10^3^ W/mm^2^, for aluminum and copper, about 1.5 × 10^4^ W/mm^2^, and for tungsten, as high as 1.5 × 10^5^ W/mm^2^—the laser absorption coefficient for all engineering materials is about 90%, regardless of the wavelength of the laser radiation [1]. At laser beam power densities above the threshold value, the laser-treated metal is immediately melted and then vaporized, which occurs in cutting, perforation, and especially ablation processes. 

## 2. Materials and Methods

**Figure 4:**
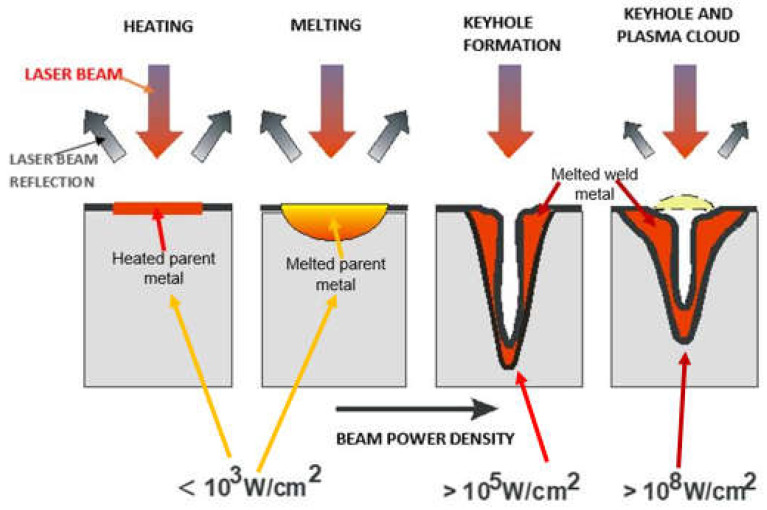
Effect of laser beam power density on relative melting depth and the shape of the absorption area of the laser beam energy on the surface of the welded joint [1,4,7,8].

**Figure 5:**
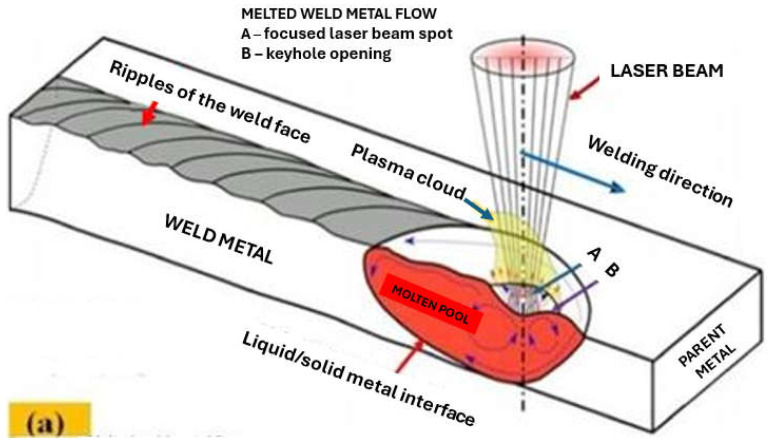

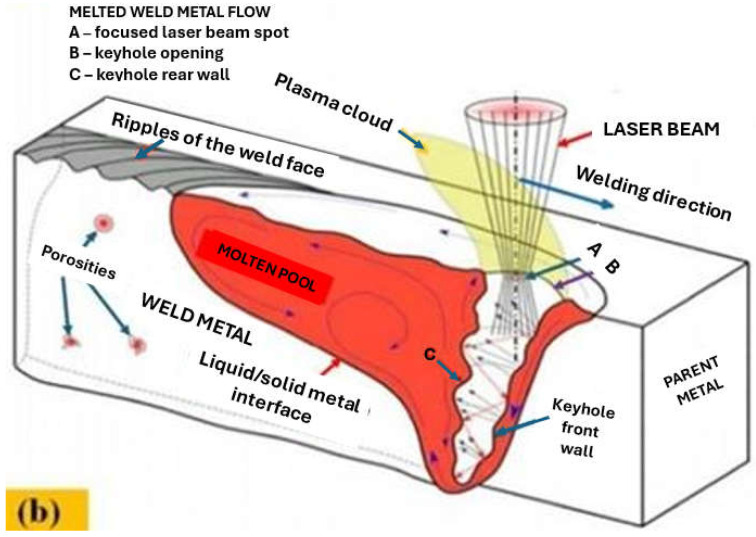
Schematic drawing of laser beam physics in laser beam welding in case of (**a**) conduction (melt-in) welding mode, (**b**) keyhole welding mode [1,21].

**Figure 6:**
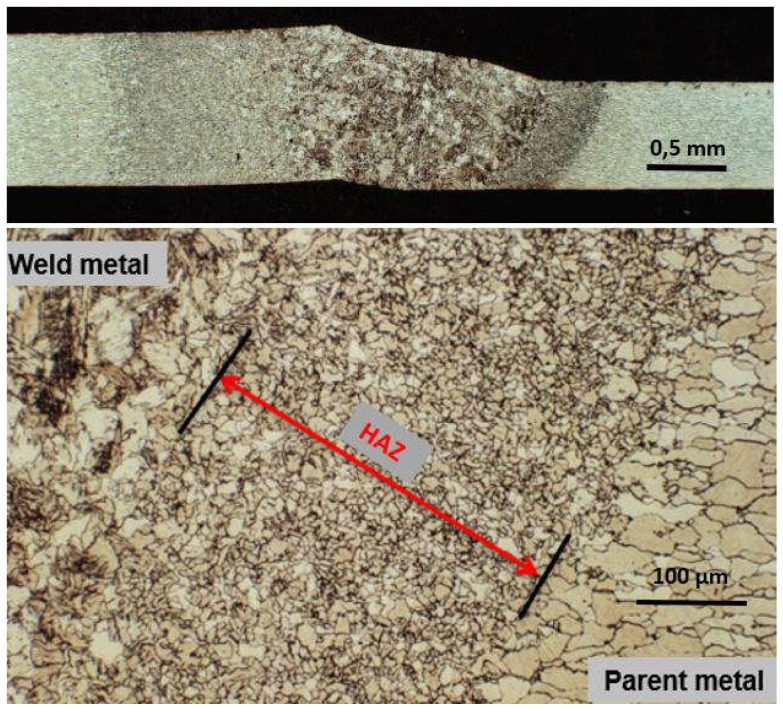
Macro- and microstructure of the butt joint of galvanized sheets, 1.2 + 0.8 mm thick, ROFIN DL 020 autogenous laser melt-in welded in a flat position. Laser beam power 1.2 kW, welding speed—0.55 m/min, gas argon shielding gas at flow rate—5.0 L/min, beam focus dimensions—6.8 × 1.8 mm [1].

**Figure 7:**
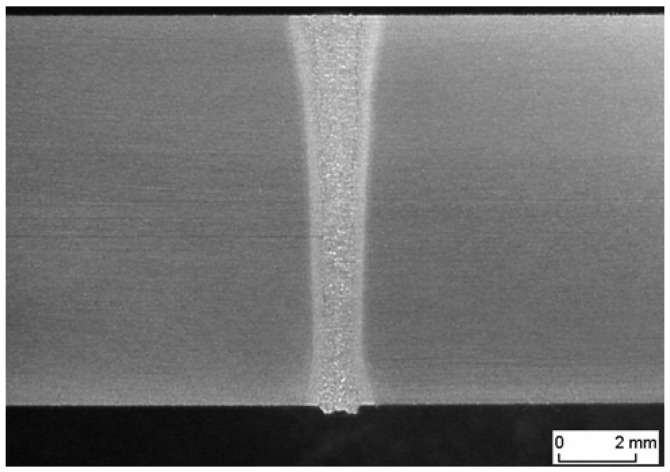
The fiber laser YLR—10.000, autogenous keyhole mode welded butt joint of API 5LX70 pipes, wall thickness of 11.2 mm. Laser beam power—10.2 kW, welding speed—2.2 m/min, shielding gas argon, flow rate—12.0 L/min [1].

**Figure 8:**
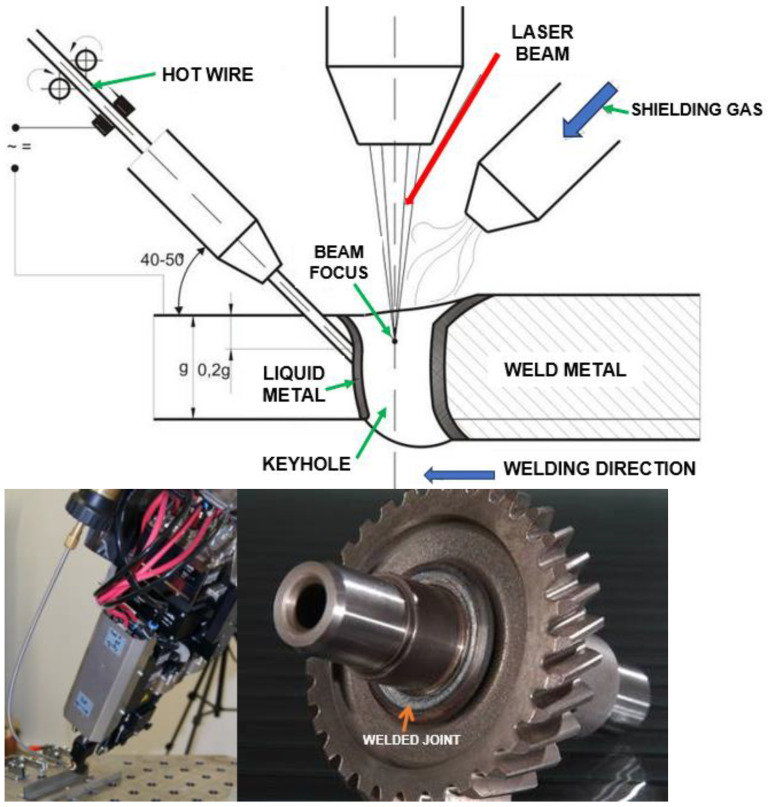
The diagram of the laser beam keyhole mode single pass welding process with hot wire feeding in the weld pool keyhole area. A view of the welding head of the TruDisk 12002 laser during the testing of the cold wire T-joint welding process and a view of the cold wire laser keyhole welded drive shaft gear joint of high-alloy tool steel [1,8].

**Figure 9:**
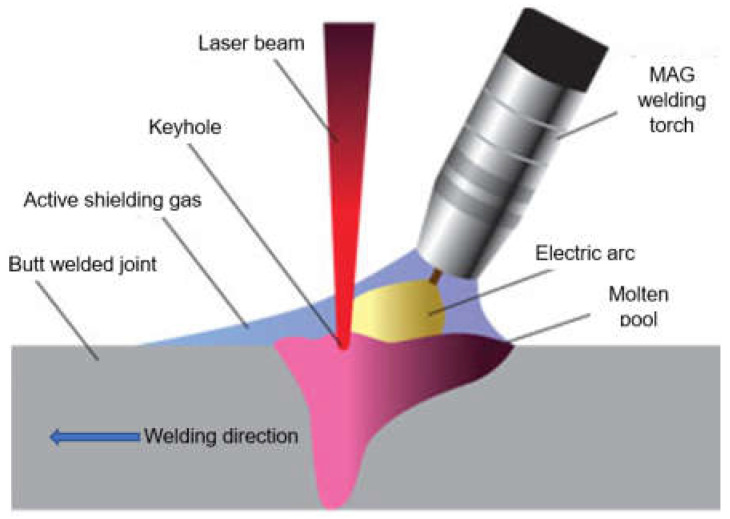
The diagram of the laser hybrid welding process: laser beam + MAG [1].

The laser beam energy can be applied for partial melting of the welded joint (melt-in mode or conduction welding mode) or melting through (keyhole welding mode) of the welded joints in the protective gas shield, as shown in Figure 4 and Figure 5. The weld metal is then formed from the melted edges of the welded materials, with the same chemical composition as the parent material, but there are significant changes in the structure of the weld metal, as shown in Figure 6 and Figure 7. Laser welding can also be carried out with the feeding of the filling material into the weld area, similar to the GTA and PTA welding processes, as shown in Figure 8, or as a so-called Laser Hybrid Welding process (LHW), which combines the highly focused intensity of a laser beam with the joint filling capability of the traditional GMA process, as shown in Figure 9. LHW is an automated or robotized high-performance welding process which results in a very narrow HAZ with deep penetration and high travel speeds, relative to such traditional welding processes as multi-wire SA or GMA. By combining the two, laser beam and GMA arc, laser hybrid welding provides a unique opportunity for thicker welded joints with less filler metal or higher travel speeds than typical arc welding processes, depending on the welded joint thickness [1,4,10,11,12,13,14,15,16,17,18,19,20,21].

**Figure 10:**
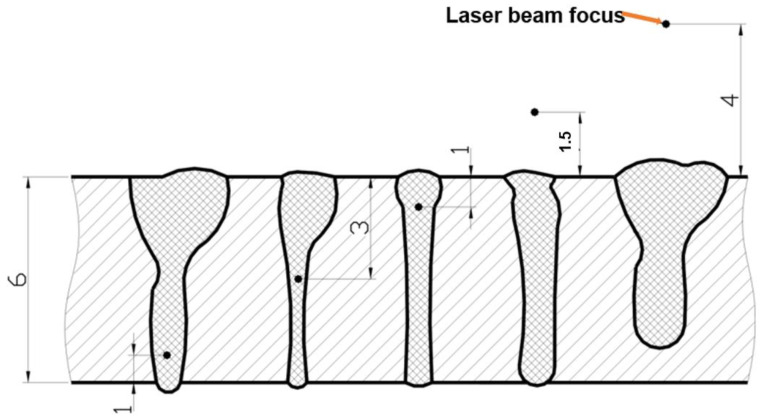
The effect of the 5.0 kW CO_2_ laser beam focus position, relative to the top surface of a 6.0 mm thick sheet of AISI 304 austenitic steel on the shape of the keyhole penetration of a weld metal in the bead on plate experiments [1].

**Figure 11:**
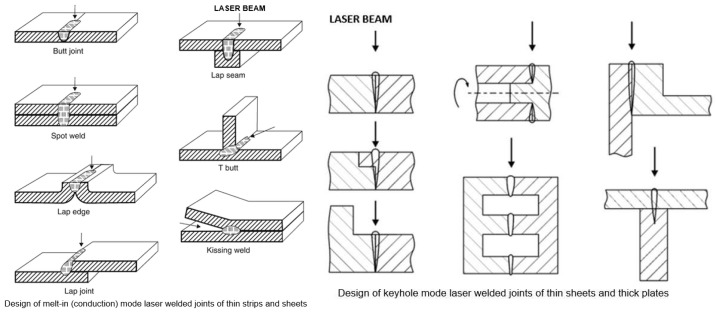
The typical design of laser-welded joints [1].

Laser welding energy (heat input) of a welded joint is produced as a result of the absorption of the energy of the concentrated beam of laser radiation by a welded joint area, with an easily regulated laser beam power density, from 10^2^ up to 10^12^ W/mm^2^. Unlike well-known arc welding processes, such as MMA, GTA, GMA, SSA, SA, or PAW, in laser welding technology, laser beam energy is derived from a laser beam generator, through a mirror or a fiber system, and a focusing lens, and is focused above the surface, on the surface, or under the surface of the welded joint, as shown in Figure 10 [1]. From the point of view of the metallurgical, technological, and structural weldability, laser welding technology provides the ability to make high-quality joints of metal and thermoplastic structures from structural materials of poor weldability, or even materials non-weldable by arc welding processes, as shown in Figure 11.

In summary, the main advantages of laser beam welding technology, which are mainly due to the ability to precisely adjust the welding heat input of the highly focused laser beam with high power density, are as follows [1,2,3,4,5,6,7,8,9,10,11,12,13,14,15,16,17,18,19,20,21,22]:

- High-quality welded joints with very narrow HAZ and minimal welding residual stresses and distortion;
- highest welding efficiency and ease of automation and robotization;
- ability to weld joints in all positions and in-field environments;
- ability to weld joints of the structures that do not require further machining or finishing;
- a very good technological, metallurgical, and structural weldability;
- limited harmful effects on the operation and the environment;
- simple staff training and operation of the equipment.

The melt-in mode laser welding is used in global industry as the more efficient and higher-quality substitution of TIG and PAW welding processes due to higher quality of joints, very narrow HAZ, and high welding speeds, as shown in Figure 6 and Figure 11. A laser beam with a beam focus shape that is circular, rectangular, or square, with a low or medium laser beam power density, of the order of 10^2^–10^4^ W/mm^2^ (depending on the type of material to be welded), is used, as shown in Figure 2. Laser beam energy heats up the upper surface of the welded area by absorption, and the subsurface area is heated by conduction. The depth of remelting of a laser-welded joint then depends on the physical properties of the welded material, mainly the absorption coefficient, but also the thermal conductivity coefficient, the melting temperature and evaporation temperature, its surface condition, and the power density of the laser beam. The melt-in mode laser welding is mainly used for autogenous one-pass welding of all types of joints, with thicknesses ranging from foils to sheets of 2.0–5.0 mm thick, depending on the type of material to be welded and its weldability. Laser melt-in mode welding can be carried out without or with filler material, single-layer or multi-layer, single- and double-side, such as in GTA and PAW welding processes, as shown in Figure 8. Two-side multilayer laser melt-in welding is recommended especially for welding butt joints of greater thicknesses, even up to 10.0–15.0 mm. To work out the WPS, it is necessary to properly select the chemical composition of the filler metal and the type of shielding gas, and often the thermal conditions of preheating, interpass temperature, and cooling speed of the joint must be controlled.

Keyhole mode laser welding is the most widely used in the industry due to the very high quality of joints, very narrow HAZ, and high welding speeds, as shown in Figure 4, Figure 5, Figure 7, Figure 10 and Figure 11. The process requires a laser beam with medium or high power density, usually above 10^4^–10^6^ W/mm^2^, and often up to 10^9^–10^12^ W/mm^2^, depending on the thickness and type of material to be welded. At the same time, the laser beam must have a circular focus, with a small diameter, on the order of 10–2000 µm, and a Gaussian distribution of TEM_00_, as shown in Figure 2. For the keyhole laser welding technique of butt joints of steel sheets up to 2.0–3.0 mm thick, the laser beam quality factor BPP (Beam Parameter Product) must be in the range of 0.3 to 1.0 mm∙mrad, and for joints up to 20.0–30.0 mm thick, the BPP must be kept in the range of 5.0–15.0 mm∙mrad. The laser beam constriction radius, w_0_, and the beam deflection angle in the far field, Θ_G_, determine the quality of the laser beam, as shown in Figure 1; BPP = w_0_∙Θ_G_ = λ/(K∙π) − mm∙mrad, where w_0_ is the radius of the laser beam constriction (diameter of the focus d_Of_), ΘG is the angle of deflection of the laser beam in the far field, λ is the wavelength of the laser beam in mm, and K is the coefficient of propagation of the laser beam (index of the quality of the beam focus) [1,3,8].

Due to the high power density of the laser beam in keyhole mode welding in the range of 10^4^–10^12^ W/mm^2^, the physical properties of the welded metal do not play as important a role as in the case of the laser melt-in welding, and, in principle, do not limit the depth of keyhole penetration. The very high power density of the high-quality TEM_00_ laser beam, with a small focus diameter (10–100 µm), with no gap between the edges of the welded joint, causes immediate melting and evaporation across the weld area of the joint and the formation of a keyhole channel, filled with weld metal vapor and gases, with the channel wall covered with a thin layer of welded liquid metal, as shown in Figure 4 and Figure 5. The laser beam energy is absorbed throughout the depth of the formed vapor channel, and as the laser beam moves along the weld line, the keyhole channel is displaced, and the liquid metal from the front wall of the channel flows in the opposite direction to the welding direction, where it solidifies to form the weld. Under the stable laser keyhole welding condition, the keyhole would stay in equilibrium among the different driving forces, such as ablation pressure, metallic vapor pressure, surface tension, gravity, and induced electromagnetic forces. The same driving forces also produce the flow of the molten pool. Once the equilibrium is broken, the keyhole would collapse. The very high power densities of the laser beam ensure that the heat inputs of laser keyhole welding are at the level of the minimum energies required to melt the joint, and the HAZ and melt zone are very narrow, while weld residual stresses and strains are at a minimum level, as shown in Figure 7 [1,7].

The basic laser beam welding parameters are as follows:

- power of the continuous wave laser beam in kW;
- laser pulse energy in kJ, pulse duration on ms, pulse repetition rate in Hz;
- the laser beam quality factor BPP;
- welding speed (laser beam travel) in m/min;
- focus length of the laser beam in mm;
- shape and dimensions of the laser beam focus in mm;
- position of the laser beam focus relative to the top surface of the joint in mm;
- type and flow rate of the shielding gas in L/min.

The power of the laser beam can be regulated:

- by changing the power of the HF electrical discharge system in CO_2_ lasers,;
- in the Nd:YAG, fiber, and disk lasers by changing the power of the flashlamp pumping system, arc, or diode laser pumping power;
- in the high-power diode lasers by changing the DC current.

The global industry manufactures of modern laser machines for laser welding applications in the steel industry are producing laser apparatuses emitting impact, pulsed, modulated, or continuous laser beams with powers up to 120 kW [1,5,6,7,8]:

- CO_2_ lasers—max. power 20 kW (TruFlow 20.000, [8]);
- fiber lasers—max. power 120 kW (YLS-120.000, [7]);
- disc laser—max. power 16 kW (TruDisk 16.002, [8]);
- diode lasers—max. power 45 kW (LDF-45.000 [5,6]).

**Figure 12:**
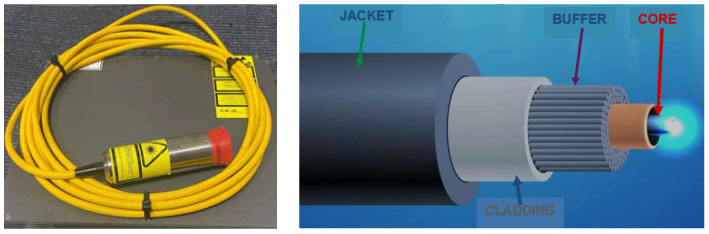
A view of the coiled fiber optic IPG DLM-200-976-EL2 LASER and design of single mode laser beam TEM_00_ fiber optic [1,7].

**Figure 13:**
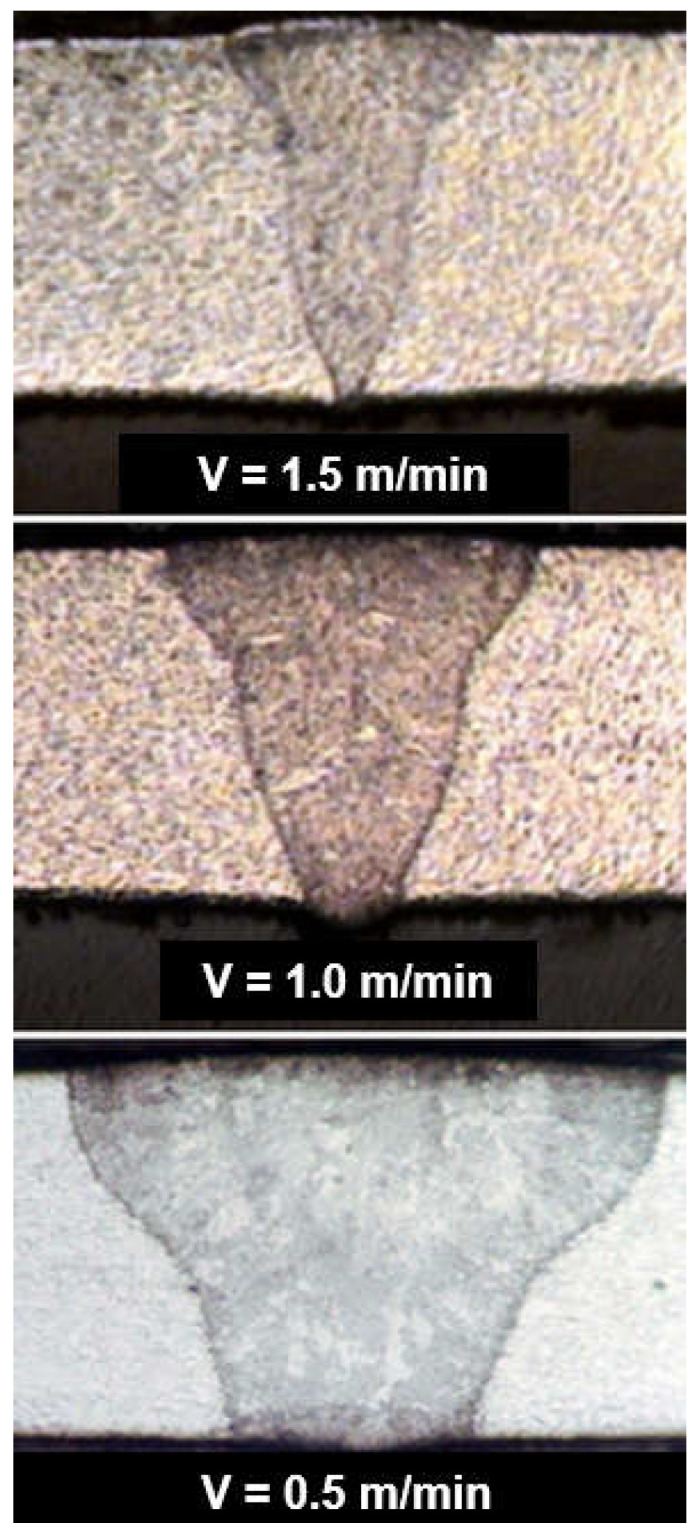
The effect of CO_2_ laser melt-in mode beam power 100 W and welding speed on the shape of the butt joint of AISI 304 austenitic steel sheets 0.5 mm thick weld metal of the butt joint. Laser beam focus diameter—10.0 µm [1].

**Figure 14:**
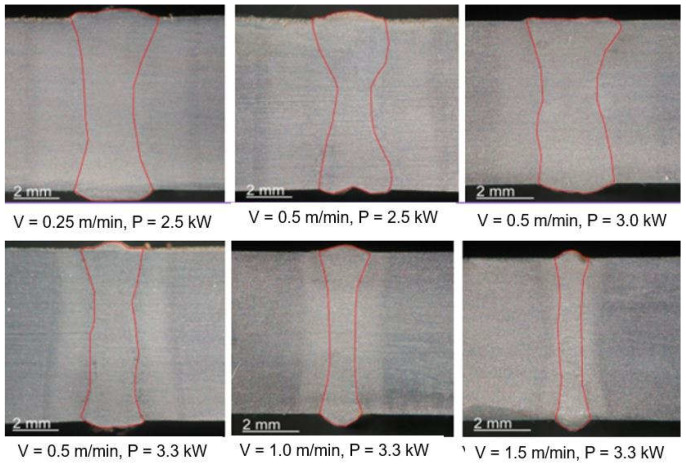
The effect of the TruDisk 3302 disk laser keyhole welding parameters, welding speed—V and laser beam power—P, on the shape and quality of the weld beads of butt welded joints of S335 steel sheets 6.2 mm thick. The TEM_00_ laser beam was focused on the top surface of the joint. Focal length—223.0 mm and focus dia.—80.0 μm. Argon shielding gas, flow rate—10.0 L/min [1].

**Figure 15:**
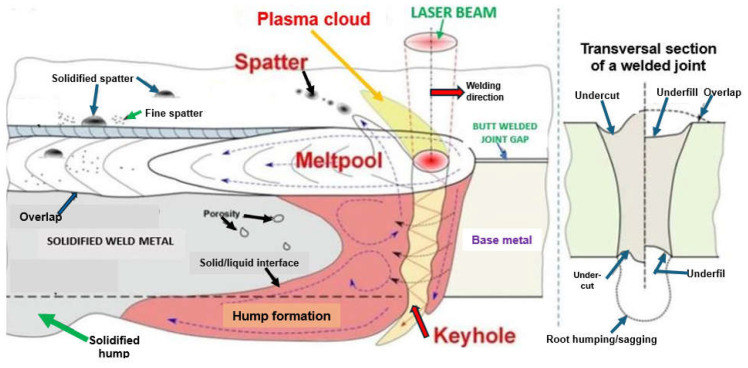
The schematic illustration of the typical welding defects of laser beam keyhole welded butt joints [1,23].

**Figure 16:**
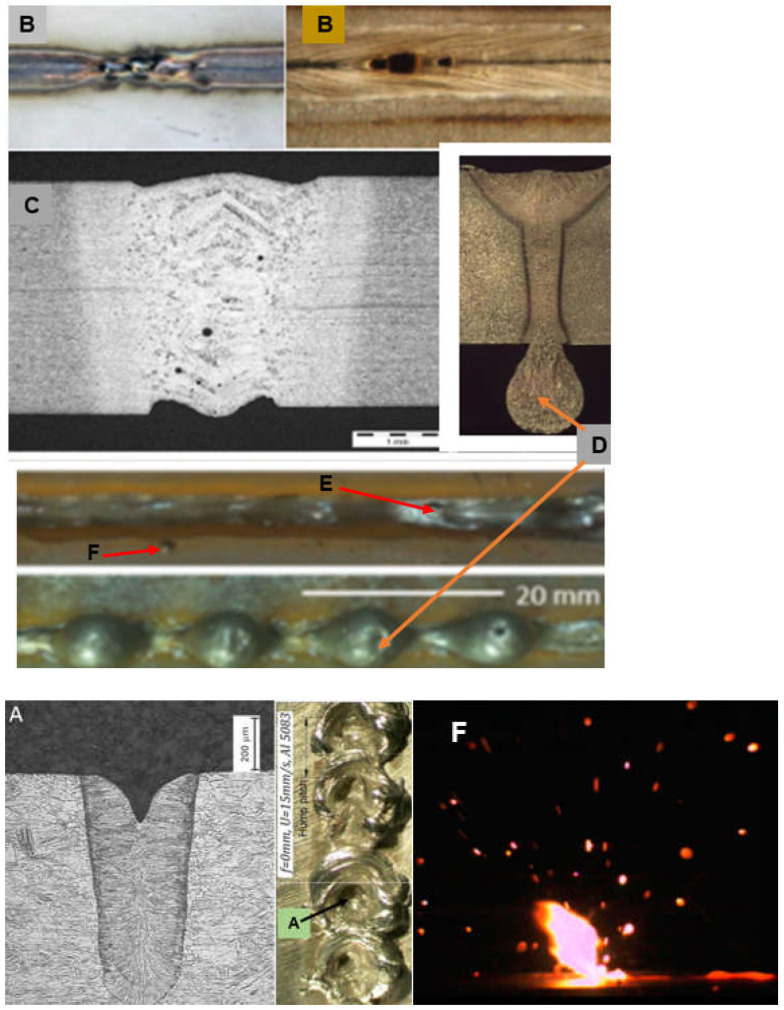
A view of typical external defects of laser-welded joints: (**A**) sharp concavity of the weld face (blow-out), (**B**) burn-through, (**C**) undercuts on the face and root side of the butt joint, (**D**) weld metal root sagging, (**E**) root humping/sagging, (**F**) metal spatter [1,23,24,25,26,27,28,29].

**Figure 17:**
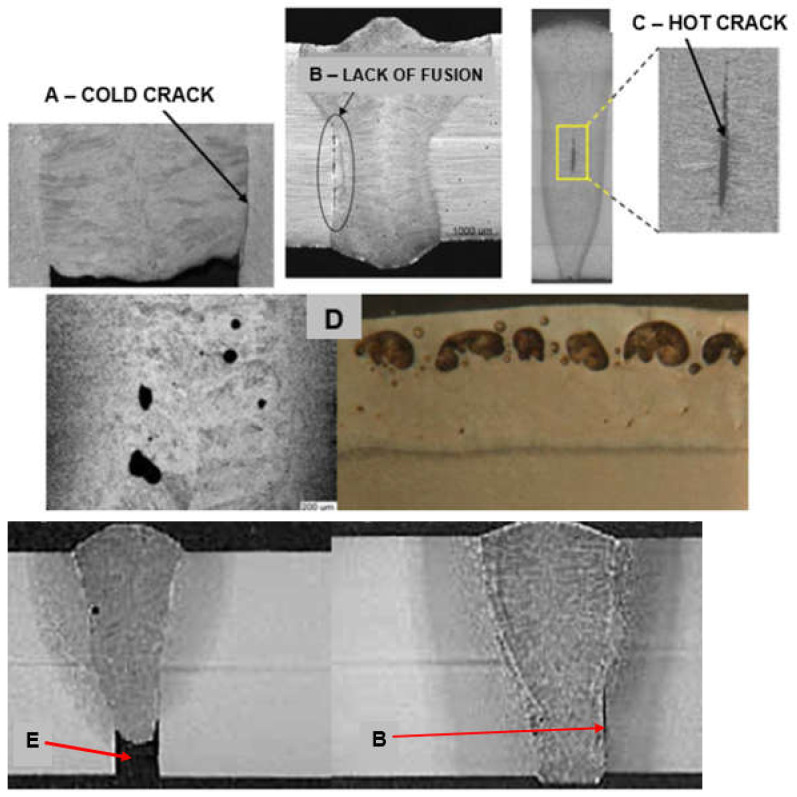
A view of typical internal defects of laser butt welded joints: (**A**) cold crack, (**B**) lack of fusion—no melting with the wall of the welded joint, (**C**) hot cracks, (**D**) porosity of the weld metal and under weld face, (**E**) lack of penetration [1,23,24,25,26,27,28,29].

The much shorter wavelength of the emitted laser beam of solid-state lasers, including fiber, disc, and diode lasers, is in the range of 450–1080 nm, and CO_2_ laser beams are in the range of 10.6 µm, showing that solid-state lasers are characterized by much higher absorption coefficients of the material being processed with the laser beam, as shown in Figure 3, and this also makes it possible to transmit a laser beam via flexible optical fibers over considerable distances, even more than 100 m, which is impossible in the case of CO_2_ lasers, as shown in Figure 12 [1,17]. It is specifically beneficial for the automatization and robotization of welding processes. In the case of melt-in mode welding, with an increase in the laser beam power density (e.g., increasing a welding speed or a laser beam power), the depth of penetration of a butt welded joint increases almost linearly, and the weld metal fusion line shape changes from a weld with an elliptical outline of the fusion line to a U-shape, as shown in Figure 6 and Figure 13. A further increase in the power density of the laser beam leads to the full penetration of the butt welded no-gap joint and the formation of a keyhole channel, with a high thickness-to-width ratio, and nearly parallel fusion lines of the weld metal, as shown in Figure 7 and Figure 14. Exceeding a certain amount of laser beam power for a given type of metal and thickness initially results in the formation of high porosity in the weld metal area, followed by concave and uneven weld face and undercuts, until the final leakage of the metal in the form of the weld metal root sagging, as shown in Figure 5, Figure 15, Figure 16 and Figure 17. To overcome the issue of the poor weldability of laser keyhole welded base material (e.g., a large number of non-metallic inclusions and strong gas saturation), and to obtain high-quality welded joints, it is advised to feed to the weld pool additional material with a precisely selected chemical composition, as shown in Figure 8. By selecting the chemical composition of the filler material for a specific type of the base metal, it is possible to eliminate the tendency to form brittle structures or intermetallic precipitates and porosity in the weld metal, often leading to welded joint cracks.

The speed of laser welding affects the weld shape and depth of penetration in a similar way to laser beam power, as shown in Figure 14. An increase in laser welding speed at constant laser beam power causes the depth of penetration to decrease for both welding modes, melt-in and keyhole. At the same time, the weld metal becomes narrower, the height of the weld face reinforcement increases, the weld “shifts” upward, the height of the root face decreases markedly, up to the occurrence of defects such as weld face and root face undercuts, excessive weld face reinforcement, and, eventually, defects of lack of penetration, as shown in Figure 15, Figure 16 and Figure 17. The outline of the weld metal fusion line also changes from oval to mushroom-shaped, and eventually with almost parallel sides. At excessive laser welding speeds, the weld metal melts and solidifies too quickly, porosity appears, and weld metal may not have time to melt parent metal, and as a result, on the upper edges of the welded joint, the weld metal is in the form of roller-like, narrow weld deposits. On the other hand, too low a laser welding speed significantly increases the width of the weld and HAZ, and in the case of keyhole welding, the weld metal leaks out of the channel and a defect of the weld metal root sagging appears, as shown in Figure 16D. In the case of laser melt-in welding, excessive reduction in the welding speed results in porosity of the weld metal (as an effect of excessive evaporation of the superheated weld metal), irregularities of the weld face, and eventually weld metal leakage, just as in the case of excess laser beam power. The highest laser welding speeds’ very narrow weld beads can be obtained when the plane of polarization of the laser beam coincides with the direction of welding. In order to be independent of the polarization of the laser beam, mostly circular polarization is used, i.e., equal in all directions, with an energy distribution at the focus of the TEM00 laser beam, as shown in Figure 2.

The dimensions of the laser beam focus depend on the quality of the laser beam BPP, which depends on the deviation of the beam in the far field Θ_G_. This divergence for laser beams of the highest quality, BPP < 5.0 mm × mrad, is usually below a few milliradians. Focusing of the laser beam is performed by high-accuracy optical systems using quartz glass or semiconductor lenses. In the case of laser keyhole welding of butt no-gap joints, as shown in Figure 5, Figure 9 and Figure 14, the laser beam must be focused to a very small possible diameter, to obtain the highest power density. These diameters range from a minimum of 0.003–0.020 mm to 0.5–2.0 mm, in the case of CO_2_ lasers and fiber and disk lasers. Modern diode lasers provide circular focus spot dia. of 0.2–0.6 mm, as well as square or rectangular focus spots ranging from 0.4 × 0.4 mm to 1.8 × 6.8 mm or up to 8.0 × 8.0 mm, or any customized shapes, as shown in Figure 2 [5,6]. When the diameter or dimensions of the laser beam focus are too small in relation to the gap width of the welded butt joint, it can result in the formation of defects of lack of fusion, lack of penetration, and concavity of the weld face. On the other hand, an excessive diameter of the laser beam focus prevents the formation of a keyhole channel in the welded joint, due to the insufficient power density of the beam and, consequently, the defect of lack of penetration, as shown in Figure 17.

**Figure 18:**
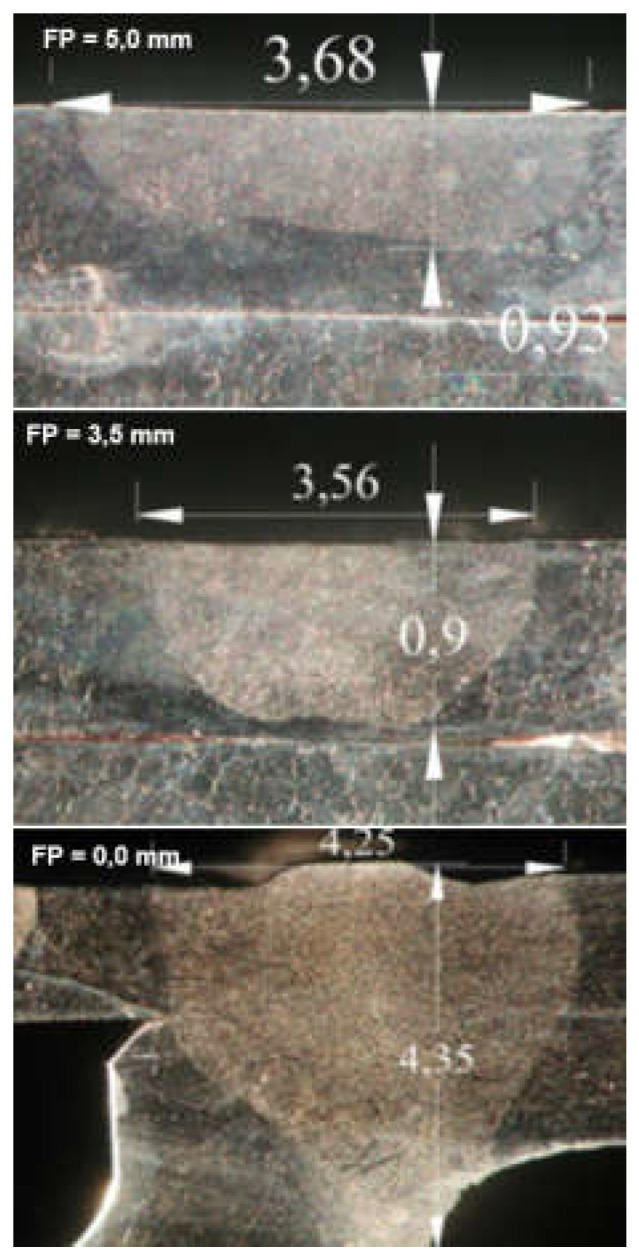
The effect of the focus position, FP, of the TRUMF TruDisk 3302 disk laser beam, relative to the upper surface of the overlap joint 1.5 + 1.5 mm thick of the nozzle apparatus ring of the turbine of the engine TW2-117 of the MI-8 helicopter, made of austenitic steel EI-835 (25% Cr, 16% Ni, 6% Mn), on the shape of the melt-in welded beads. Laser beam focus diameter—80 µm, focal length—223.0 mm, beam power—1.4 kW, welding speed—0.3 m/min, argon shielding gas, flow rate—8.0 L/min [1].

The position of the laser beam focus relative to the top surface of the laser-welded joints determines the shape and depth of the welded joint penetration, as shown in Figure 9, Figure 10 and Figure 15. Any change, even small, in the position of the laser beam focus over or below the top surface of the welded joint causes the absorption area of the laser radiation to increase, so the real power density of the laser beam decreases and the shape of the penetration changes. The higher the quality of the laser beam (minimal BPP), the smaller this change is, due to the smaller deflection angle of the laser beam in the far field, Θ_G_, as shown in Figure 1. The position of the laser beam focus in the laser melt-in welding process has a lesser effect on the weld bead shape than in keyhole welding, mainly due to the much lower laser beam power densities used in the melt-in laser welding processes. Changing the position of the laser beam focus above and below the surface of the welded joint in both cases increases the weld bead width and decreases the penetration depth, as shown in Figure 10 and Figure 18. Thus, in the process of laser melt-in welding, it is usually recommended to focus the laser beam on the top surface of the welded joint. The optimal shape and high quality of laser keyhole welded joints are ensured when the position of the laser beam focus is on the top surface of the joint or inside the joint, at a depth equal to 10–30% of the welded joint thickness. For high-quality laser beams (TEM_00_ and minimal BPP) and welded joint thicknesses up to about 5.0–6.0 mm, it is recommended to focus the laser beam on the top surface of the joint, and for thicknesses above 6.0 mm, in the range of 1.0 to 3.0 mm below the top surface of the joint [1,5,6,7,8,9,22].

The double Rayleigh length (depth-of-focus) of the laser beam, ZR, is directly proportional to the length of the focus, as shown in Figure 1. Optical systems with a focus length of the order of 38–75 mm provide greater depths of penetration, but then the shape of the weld bead and the depth of penetration largely depend on the accuracy of maintaining a constant focus position. Increasing the focus length from about 125 mm to as much as 2500 mm allows for greater tolerance of the laser beam guiding system along the laser welding line and for variations in the distance of the welded joint from the laser head’s nozzle. This is particularly useful when welding joints with corrugated surfaces or irregularities and is used in scanning robotized welding systems. In the case of CO_2_ lasers, the shortest allowable focal distance is 125 mm, due to the risk of damage to the optics by metal vapors emitted by the weld pool and weld metal spatter. The passage of the CO_2_ laser beam into the welding area involves only minor losses of laser beam energy on guide elements, mirrors, apertures, and optics and, as a result of gas ionization in the air, in the plasma plume above the laser welding area, as shown in Figure 5 and Figure 15. Laser beam energy losses on the fiber optics transporting a laser beam from the solid-state laser generator to the welding area are on the order of 5–20%, depending on the power of the laser beam and the length and type of material of the optical fiber, as shown in Figure 12. Since the size of the focus of the laser beam is inversely proportional to the diameter of the laser beam entering the laser head optics, special optics are used to expand the laser beam. This makes it possible to use larger focal lengths without losing power density.

**Figure 19:**
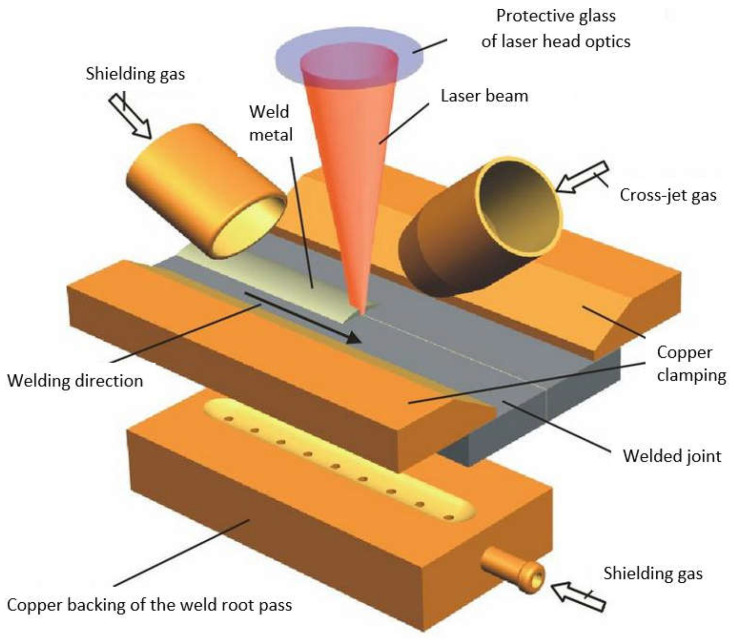
The schematic diagram of gas shields of laser welding processes [1].

**Figure 20:**
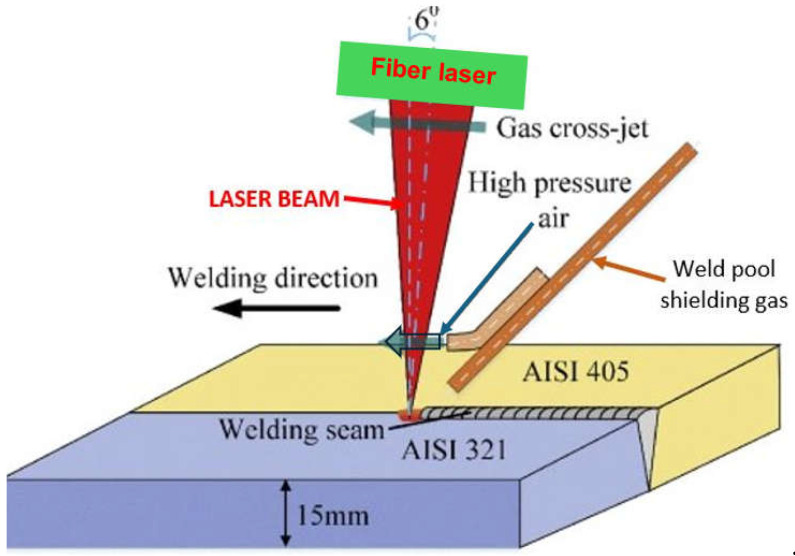
The schematic diagram of the arrangement of high-power laser keyhole welding of thick plates with air cross-jet application [1,20].

When the energy of the laser beam radiation is converted to the welding heat, resulting in the melting and vaporization of the welded joint’s metal, a plasma plume of ionized metal and gas vapors forms over the liquid weld pool, as shown in Figure 5 and Figure 15. This plasma plume strongly absorbs the energy of the laser beam, thus lowering the power density, which, as a result, can lead to the lack of formation of a proper keyhole channel, leading to the formation of defects of lack of fusion and lack of penetration, as shown in Figure 17. For this reason, it is necessary to blow the plasma plume away from above the welding area by means of cross-jet flow of protective gas to the laser beam axis, just below the laser head, so that it does not interfere with the shielding gas flow of the weld pool, but at the same time effectively blows out welding fumes and vapors and liquid metal spatter droplets from above the weld pool, as shown in Figure 19 and Figure 20. Typical cross-jet gas for the laser welding of steel joints is air or the same gas as weld pool shielding gas, such as He, Ar, Ar + CO_2_, or N_2_, as shown in Table 1. The other task of the cross-jet gas is to protect the optical system of the laser head from metal spattering from the welding area. The best results are provided by blowing the plasma plume with helium or a mixture of He + Ar, since the high ionization potential of He reduces the amount of plasma gas produced.

Pulsed-beam or continuous-beam laser welding with low- or medium-power lasers requires the gas shielding of the weld pool, in addition to the cross-jet gas, to remove the plasma plume from above the welding area. For laser powers up to 5.0 kW, argon, helium, or mixtures of them can be used as a weld pool shielding and plasma plume removing gas. In high-power CO_2_ gas laser welding processes, it is recommended to blow helium protective gas transverse to the laser beam axis and in the direction of welding, and to use additional gas shielding of the weld root pass area, as shown in Figure 19. Sometimes, double gas blowing is recommended, first at the top surface of the joint, with an inert gas, and second above, using something like an air stream to protect the laser optics. In the case of laser welding of alloyed and high-alloy steel joints, the recommended shielding gases are mixtures of argon+20–30% CO_2_ or pure CO_2_. The flow rate of the shielding gas should be in the range of 10–12 L/min. In laser welding of thin sheets of low-carbon steel, with a thickness of less than 3.0 mm, thanks to high welding speeds of more than 2.0–3.0 m/min and very short residence times of the liquid weld metal at oxidation temperatures, it is possible to completely dispense with the welded area shielding gas and the gas blowing out the plasma plume, and apply only the cross-jet air nozzle, to protect the optical system of the laser head.

Basic restrictions of the laser welding technologies are as follows:

- the high cost of the laser equipment and automatic or robotic stands in comparison to classic welding processes;
- poor weldability of structural materials with low laser radiation absorption, such as copper, aluminum, magnesium, and their alloys, as shown in Figure 3;
- requirement of the high precision of the laser beam focus along the welding track and the high accuracy and purity of the preparation of joints for laser welding processes.

In addition, it is necessary to ensure high stability of the basic welding parameters, in particular, the laser beam power and welding speed, the position of the laser beam focus relative to the surface of the welded joint, the focal length (dimensions of the laser beam focus), and the flow rate of the shielding gas. Even a slight change in any of these welding parameters may lead to the formation of usually unacceptable external defects in welded joints: irregular and concave weld face, underfilling, undercuts, burn-through, weld sagging, metal spatter, and internal defects, including very dangerous and difficult-to-detect internal lack of fusion (especially when welding is carried out by keyhole mode welding), cold cracks and hot cracks, and porosity, as shown in Figure 15, Figure 16 and Figure 17 [1,4,10,11,12,13,14,15,16,17,18,19,20,21].

**Figure 21:**
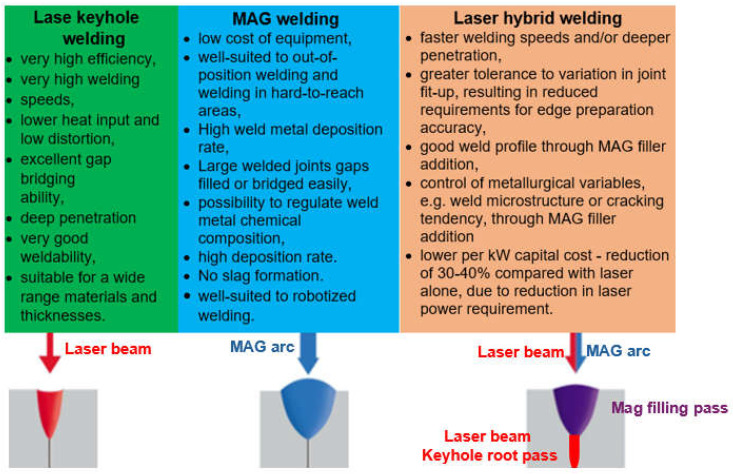
A comparison of advantages of laser keyhole welding, MAG welding, and laser + MAG hybrid welding [1].

**Figure 22:**
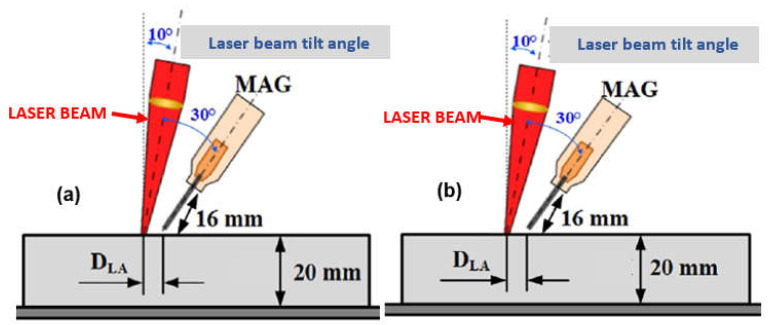
A view of the two techniques of the ultra-high-power LHW process: (**a**) laser beam leading mode, (**b**) MAG arc leading mode [1,18].

**Figure 23:**
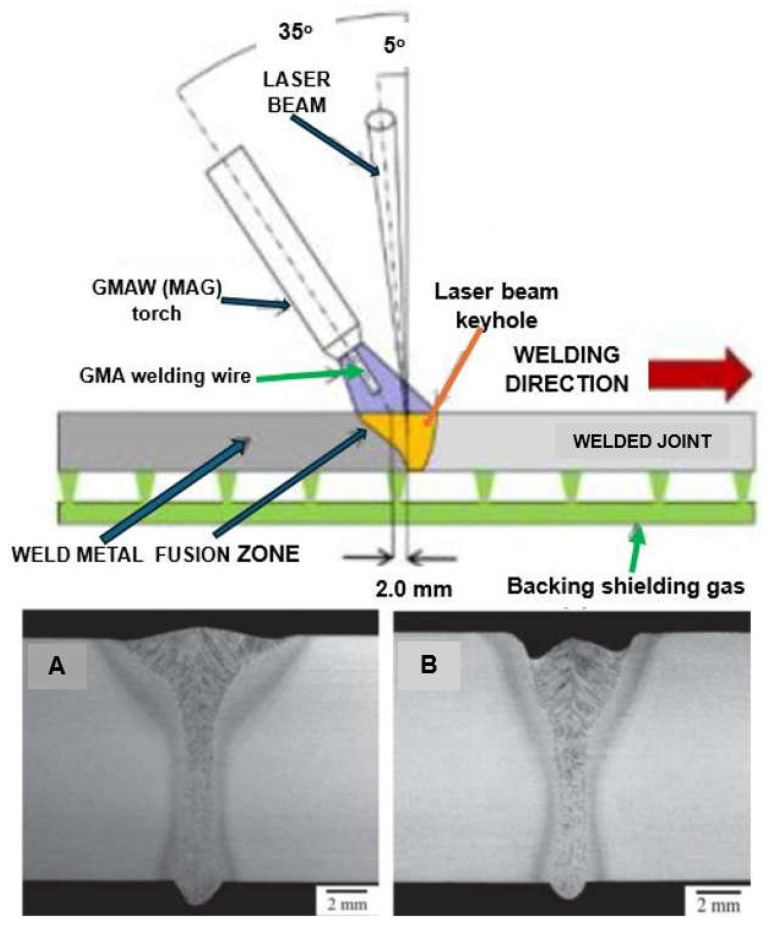
A schematic diagram of the LHW process with LASER beam leading mode. Welded joint macrostructures: (**A**) laser beam leading LHW, (**B**) MAG arc leading LHW [1,20].

**Figure 24:**
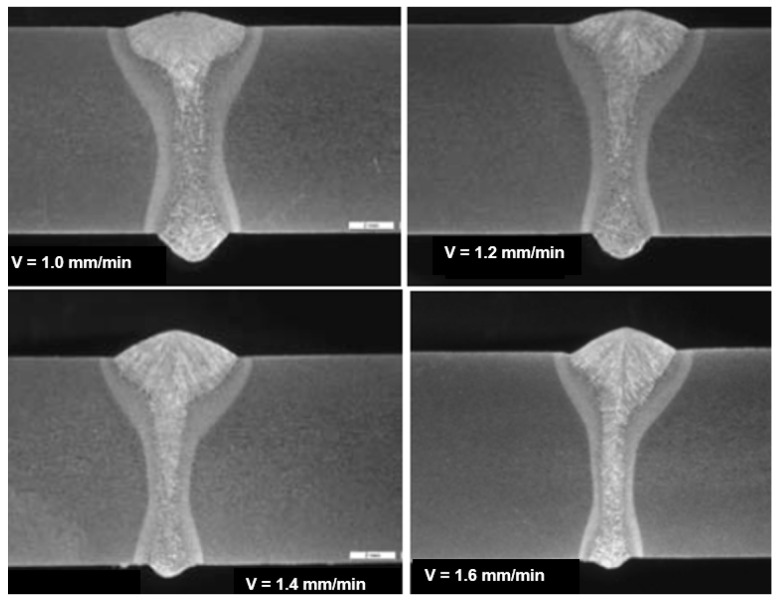
The effect of welding speed—V mm/min, on the shape of the LHW butt joints of API 5LX65 steel pipes, 9.5 mm thick. Fiber laser beam power—7.6 kW, and focus position—2.0 mm, below the top surface of the joint. Welding parameters of the filling pass MAG (DC+) welding: solid wire Nertalic 70S, SAF DUAL 200—diameter 1.6 mm, wire feed speed—6.5–9.0 m/min, wire distance from the laser beam—3.0 mm, shielding gas—Ar + 10% CO_2_, flow rate—20.0 L/min [1].

**Figure 25:**
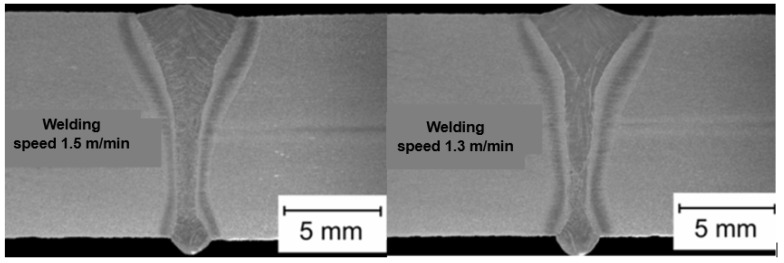
Macrostructure of LHW welded butt joints of EH36 marine low-alloy steel plates, 12.0 mm thick. Welding parameters: fiber laser YLR—10,000, beam power—8.0 kW, beam diameter—0.4 mm, focus position below the top surface of the plate—8.0 mm, ESAB Aristo power source—MAG (DC+) welding, solid wire 1.2 mm. Welding speed—1.5 m/min, wire feed speed—9.0 m/min. Welding speed—1.3 m/min, wire feed speed—10.0. For both welding speeds, the arc voltage is 33.0–33.8 V, the wire distance from the laser beam is 3.0 mm, the shielding gas is Ar + 18% CO_2_, and the flow rate is 20.0 L/min [7].

**Figure 26:**
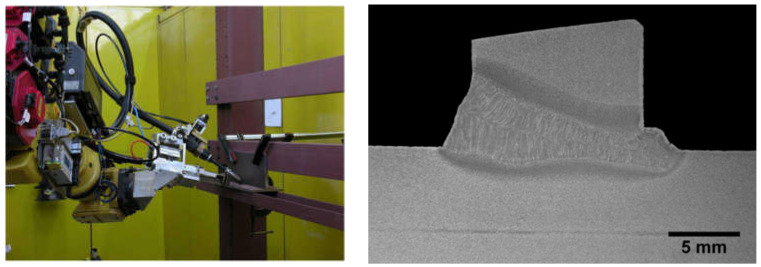
A view of the experimental stand of the LHW process of T-joint of EH 36-grade steel plates 12.0 mm thick and the welded joint macrostructure [12].

The most innovative of laser welding industrial applications (and still in development) is the laser hybrid welding process, in which a laser beam heat source and a GMA arc heat source are combined, as shown in Figure 21, Figure 22, Figure 23, Figure 24, Figure 25 and Figure 26. The Laser Hybrid Welding process (LHW) was developed to combine the advantages of the laser keyhole welding technique and the arc welding processes of GTA, GMA, and PTA (PAW). During laser beam deep penetration welding, a keyhole is generated in the molten pool of the no-gap root pass of a welded joint. During the most popular LHW process, laser beam + GMA arc, the GMA arc metal droplet would transfer to the molten pool of the laser beam keyhole and flow together as the one molten pool of the LHW process. In the LHW process, the laser beam heat source is the main factor of deep penetration of the no-gap root pass, and the GMA (or Metal Active Gas—MAG) process determines the width and depth of the filling face bead of the welded joint, as shown in Figure 23, Figure 24, Figure 25 and Figure 26.

The LHW process combines the advantages of laser beam welding with arc welding (mostly the GMA welding process). This hybrid welding approach offers many benefits, making it a popular choice in various industries producing welded metal structures, specifically high-strength steel (HSS) and aluminum alloys. These are some of the critical benefits of the LHW process compared to arc welding methods of girth-welded pipeline joints [1,4,10,11,12,13,14,15,16,17,18,19,20,21]:

- Allows for higher welding speeds. Combining laser beam and GMA arc welding creates a synergistic effect that enhances the overall process efficiency.
- The focused nature of the laser beam enables precise and efficient energy delivery to the LHW welded joint, assuring high energy efficiency. This results in reduced energy consumption per unit of LHW welded joint.
- The laser beam-focused and intense heat source results in a narrow, deep weld seam of the no-gap root pass with minimal HAZ. It leads to improved LHW welded joint quality and metallurgical and technological weldability, reduced distortion, and better control over the LHW process.
- The deep penetration capabilities of LHW make it suitable for welding in one-pass thicker joints, up to 15.0 mm in the case of (U)HSS, titanium, and aluminum alloys.
- LHW can generate fewer welding fumes, contributing to a better work environment and improved operator safety.

The LHW process has many industrial applications but is mainly limited to the workshop production of structures of various metals and alloys. Still, there needs to be documentation about in-field applications, such as, e.g., welding of (U)HSS pipeline butt joints [21]. Although the LHW has numerous process advantages, it has certain limitations too, such as very high initial investment, requirement of accurate positioning of the laser head and GMA torch, proper part fit-up of welded joints, controlling large numbers of LHW process parameters, and the requirement of additional safety measures against the laser radiation. The most common solution of the LHW welding of low-alloy and (U)HSS steels combines the laser keyhole and MAG welding processes with solid wire or flux or metal powder-cored wires [1,4,10,11,12,13,14,15,16,17,18,19,20,21]. The LHW process combines the speed and quality of laser keyhole welding with the ability to adjust the weld metal’s chemical composition by adequately selecting the MAG chemical composition of a consumable wire. As a result, higher process stability, higher welding speed, lower MAG wire consumption, and better mechanical properties of the joints are obtained than in classical laser welding and MAG welding, as shown in Figure 24, Figure 25 and Figure 26.

In the case of the butt joint welding of low-alloy and (U)HSS steels, it is recommended to prepare joints on a Y, with the height of the root face at 2.0–10.0 mm, with a gap of 0.0 to 1.0 mm, a bevel angle of 20–40°, and a bevel depth of 20–30% of the welded joint thickness, to accommodate the filler metal of the MAG process, and to ensure a smooth and even weld face, with no undercuts, as shown in Figure 24, Figure 25 and Figure 26. According to the relative position of the laser and arc along the welding direction, there are two modes: laser beam leading and MAG leading, as shown in Figure 22 and Figure 23. The LHW heat source configuration plays a crucial role in the quality of the LHW process. Laser leading mode expands the cross-sectional plasma area and thus provides a relatively stable welding process, ensuring much deeper keyhole penetration, compared to the MAG leading mode, and ensures the practical function of gas shielding while leading to a relatively wide and smooth surface formation. On the other hand, the study of ultra-high-power fiber laser 30.0 kW—IPG YLS–30.000 CW, LHW welding of butt joints of mild steel Q235-type plates, 20 mm thick, with MAG leading mode and laser leading mode, indicated that relatively stable arc characteristics were formed in MAG leading mode when compared with MAG leading mode. An enlarged laser beam keyhole, with MAG leading mode, reduces the fluctuation of metal vapor and the height of the keyhole liquid column and thus forms a stable plasma jet and the small droplet transfer of the MAG arc. At the same time, the stability of the LHW process is improved [18].

The LHW process is affected by the leading positions of the laser beam and MAG arc and the distance between the laser beam focus and MAG arc–DLA, as shown in Figure 22 [1,18]. In the LHW process, the thermal radiation effect of the laser beam plasma on the MAG droplet and the absorption effect of the laser beam plasma on the MAG arc change the shape of the MAG arc and the corresponding stress state of the droplet, which changes the droplet transfer process. Under the optimal DLA, the droplet transfer mode is a single, stable spray transfer for different welding currents, and the weld formation is smooth. Increasing the DLA could optimize the melt flow state in the MAG leading mode. However, the small DLA was preferred in the laser leading mode, since the excessive DLA would reduce the previous LHW process effect. The increase in DLA in MAG leading mode and laser leading mode, electric signals, and HSI observations revealed that the leading mode and DLA significantly affected the weld metal morphologies, process stability, droplet transfer, and defect formation. Adjusting DLA in the 2.0–4.0 mm range achieved a stable LHW process and sound welded joint formation for both leading modes [4,13,14,15,16,17,18].

## 3. AI-Supported Online Quality Monitoring

**Figure 27:**
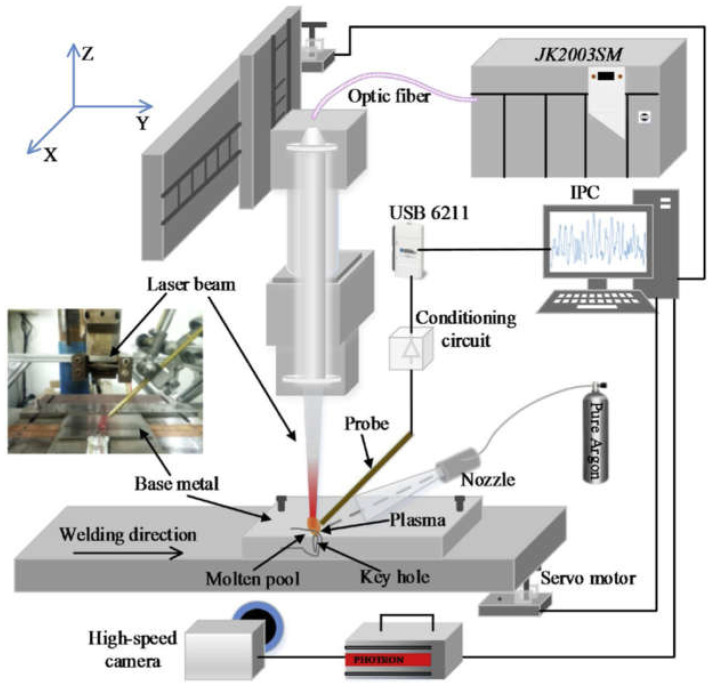
The schematic diagram of the experimental system of data acquisition of laser hybrid welding process employing a passive probe made of copper to acquire the electrical signal of laser-induced plasma, the high-speed camera (FASTCAM Super 10 KC, PHOTRON) serving to observe the dynamic behavior of plasma plume with a frame rate of 5000 fps [35].

**Figure 28:**
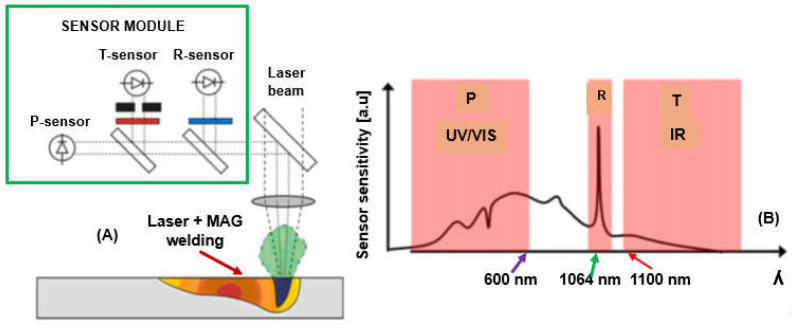
Typical laser keyhole welding online quality monitoring system using photodiode sensors for electromagnetic radiation registration: (**A**)—the system of photodiode sensors and optical filters recording the radiation of the welding area, (**B**)—the course of changes in the radiation intensity of three photodiode sensors, P—plasma cloud radiation: ultraviolet and visible radiation (UV/VIS) of 200–750 nm, R—laser beam reflection radiation, T—infrared radiation, wavelength 1100–1700 nm, Figure 29 [23].

**Figure 29:**
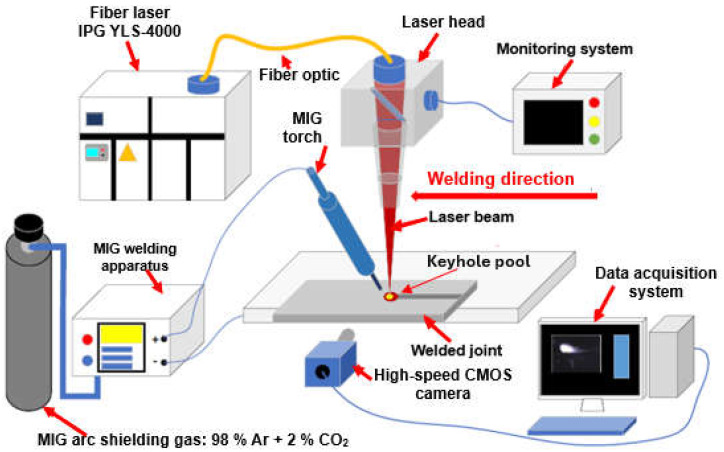
The schematic diagram of the experimental system of high-power LHW MIG leading process of AISI 304 steel sheets 4.0 mm thick for quality monitoring and weld defect detection using a high-speed imaging system that could observe the visual information from the top and bottom of the welded joint simultaneously [40].

**Figure 30:**
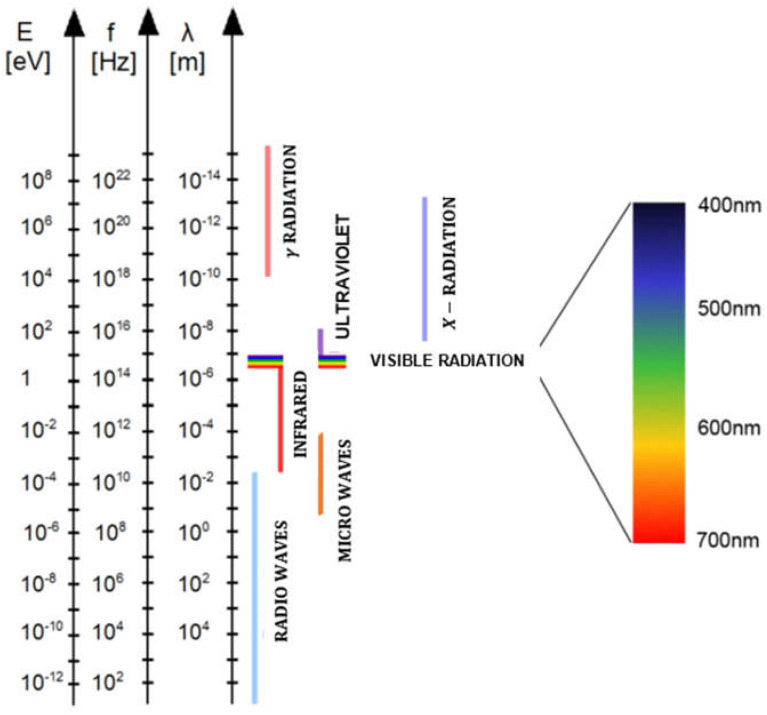
The range of energy—E eV, frequency of radiation—f Hz, and the length of the primary electromagnetic waves—λ m. Laser radiation used in welding is in the wavelength range from approx. 800 nm to 10.6 µm [1].

For the highest quality of laser-welded joints, it is necessary to maintain high stability of laser welding parameters using online welded joint quality monitoring systems and to provide their online correction, as shown in Figure 27, Figure 28, Figure 29 and Figure 30 [23,24,25,26,27,28,29,30,31,32,33,34,35,36,37,38,39,40]. In the event of changes in the quality factors, such as irregularities and contaminations of welded joint surfaces or changes in the geometry of the joint or the welding track, AI-supported online correction assures high quality of the welded joints. These AI-supported online quality monitoring systems use different sensors that record data or signals of dynamic changes in the physical phenomena occurring in other areas of laser-welded joints: a keyhole steam channel (gas cavity) of a weld bead, a cloud of plasma and metal vapor above the keyhole, or the weld pool of welded joints. Laser-welded joints emit electromagnetic radiation in an extensive range and consist of ultraviolet radiation, radiation in the visible light band, radiation generated from a cloud of plasma and metal vapor over the keyhole welding zone, the emission of solid-state or gas lasers and light reflected from the welding area and thermal radiation, infrared, and near-infrared, and additionally, acoustic sound emission, as shown in Figure 27, Figure 28, Figure 29 and Figure 30. Electromagnetic radiation generated in laser welding is the source of signals of specific quality characteristics of the welding area. The light spectrum from a laser welding zone depends on the particular laser parameters involved and the type of welded material. Still, it looks similar to the schematic for most metals during Nd: YAG laser keyhole welding. Online quality monitoring systems, integrated with laser heads, containing acoustic sound sensors, photodiode, optical (high-speed cameras), spectrometers, pyrometers, plasma plume electrical charge sensors, passive probes, etc., provide a large amount of data in the form of signals or images of the laser welding area, integrated into adaptive control systems using artificial intelligence, machine learning, neural networks, and fuzzy logic, assuring modern online quality control of welded joints. The results of the application of the AI-supported online quality monitoring systems of LBW and LHW processes indicate that it is possible to provide dynamic detection of welded joint defects and dynamic feedback control of welding parameters, to reduce or eliminate the occurrence of welded joint defects and assure precise penetration control of butt welded joints in specific [23,24,25,26,27,28,29,30,31,32,33,34,35,36,37,38,39,40].

## 4. Summary

More than 100 years ago, in 1917, Albert Einstein published a theoretical paper on possibly stimulating the emission of coherent, monochromatic electromagnetic radiation. After only 41 years, Dr. L. Maiman built the first ruby laser, emitting laser radiation with a power of several watts [40]. At the same time, all arc welding processes (but MMA) were in the first stage of development. In the last 10–15 years, laser beam industrial applications in welding engineering have enormously increased, and nowadays, solid-state lasers of power over 100 kW are available on the world markets. Laser welding is the most modern welding technology of low-alloy and high-alloy steels, titanium alloys, nickel and cobalt superalloys, and in a limited range of aluminum and copper alloy structural joints (due to the very low absorption coefficient of laser beam energy) [1,5,6,7,8,9].

The presented work is a state-of-the-art literature review and analysis and author research results on the Laser Beam Welding (LBW) and Laser Hybrid Welding (LHW) processes and industrial applications, specifically the melt-in-mode and keyhole mode laser welding techniques. The influence of basic LBW and LHW parameters on the quality of welded joints of metal structures, specifically steel structures, is thoroughly analyzed. Furthermore, the optimal LBW and LHW welding conditions in the workshop environment of low-alloy and high-alloy steel welded metal joints are listed. The severe challenge of welding engineers involved in modern metal structure production is to design and develop WPSs of LBW and LHW processes to be applicable in the harsh environments of the in-field welding of metal structures, e.g., pipelines, wind towers, offshore rigs, tanks, refinery installations, etc.

The review of the newest online quality monitoring systems of the LBW and LHW processes indicates that real-time detection and control of welding parameters and the image, plasma plume, and acoustic emission signals of a welded joint influence on the quality of welded joints during laser welding processes are far from perfect, even under laboratory experimental conditions [23,24,25,26,27,28,29,30,31,32,33,34,35,36,37,38,39,40]. However, the primary research objective should be to determine the welded joint defect detection accuracy by the newest multiple sensors of AI-supported online quality monitoring systems. The computing speed of intelligent signal processing and recognition technology also restricts the wide use of real-time detection. The results of the contemporary applications on the laboratory and industrial level of the AI-supported and digital twin-assisted online quality monitoring systems of LBW and LHW processes indicate that it is possible to provide dynamic detection of welded joint defects and dynamic feedback control of welding parameters, to reduce or eliminate the occurrence of welded joint defects and assure precise penetration control of the butt welded joints in specific. However, it is still a great challenge to design and develop an online quality monitoring system for welding processes in harsh environments such as in-field welding technologies [21].

## Figures and Tables

**Table 1:** Basic physical properties of shielding gases recommended for laser welding.

Basic Physical Properties	Ar	He	CO_2_	H_2_	N_2_
Density—kg/m^3^	1.78	0.178	1.98	0.098	1.25
Ionization energy—eV	15.7	24.9	14.4	15.4	14.5
Energy of dissociation—J/mol	—	—	2.8 × 10^5^	4.3 × 10^5^	9.4 × 10^5^
Heat capacity—J/mol × °C	21	21	60	35	—
Coefficient of thermal conductivityat 6000 K—W/m × °C	0.17	1.5	5 × 10^−5^	2.0	—
Temperature of boiling point—°C	−185.5	−268.9	−78.9	−259	−196
